# Immune checkpoint inhibitors in cancer therapy

**DOI:** 10.7555/JBR.31.20160168

**Published:** 2017-05-15

**Authors:** Eika S. Webb, Peng Liu, Renato Baleeiro, Nicholas R. Lemoine, Ming Yuan, Yaohe Wang

**Affiliations:** 1. Center for Molecular Oncology, Barts Cancer Institute, Queen Mary University of London, EC1M 6BQ, UK; 2. Sino-British Research Centre for Molecular Oncology, National Center for International Research in Cell and Gene Therapy, Zhengzhou University, School of Basic Medical Sciences, Academy of Medical Sciences, Zhengzhou University, Zhengzhou, Henan 450002, China.

**Keywords:** checkpoint inhibitor, CTLA-4, PD-1, immunotherapy, cancer

## Abstract

In recent years immune checkpoint inhibitors have garnered attention as being one of the most promising types of immunotherapy on the horizon. There has been particular focus on the immune checkpoint molecules, cytotoxic T-lymphocyte antigen-4 (CTLA-4) and programmed cell death protein 1 (PD-1) which have been shown to have potent immunomodulatory effects through their function as negative regulators of T cell activation. CTLA-4, through engagement with its ligands B7-1 (CD80) and B7-2 (CD86), plays a pivotal role in attenuating the activation of naïve and memory T cells. In contrast, PD-1 is primarily involved in modulating T cell activity in peripheral tissues via its interaction with PD-L1 and PD-L2. The discovery of these negative regulators of the immune response was crucial in the development of checkpoint inhibitors. This shifted the focus from developing therapies that targeted activation of the host immune system against cancer to checkpoint inhibitors, which aimed to mediate tumor cell destruction through the removal of coinhibitory signals blocking anti-tumor T cell responses.

## Introduction

The rapidly growing field of cancer immunotherapy has developed largely as result of our increased understanding of the immune system and malignancy^[1–2]^. One of the early developments in this field occurred when Thomas and Burnett proposed that tumor cells could evoke an immune response and this formed the basis of further research^[3]^. Following this discovery, the mechanisms of various immune cell responses involved in cancer recognition and elimination; including Forkhead box P3 (*FOXP3*^+^) regulatory T cells (T-regs)^[4–5]^, antigen-presenting cells (APCs)^[6–7]^ myeloid-derived suppressor cells (MDSCs)^[8]^ and effector T cell subsets^[9–13]^ have been elucidated.

Another important discovery in the development of checkpoint inhibitors (CIs) is the knowledge that T cell activation requires two signals. The first signal involves specific antigen recognition by lymphocytes and a second co-stimulatory signal is required as well as the existence of negative coinhibitory (costimulatory) signals, while IL-12 and type I IFN (IFNα/β) are the major sources of signal 3 in a variety of T cell activation^[14]^. Receptors such as cytotoxic T-lymphocyte antigen-4 (CTLA-4) which supply these coinhibitory signals function as immune checkpoints which play an important role in the termination of immune responses following antigen activation; essentially in the maintenance of peripheral tolerance and autoimmunity. Tumours may exploit these immune checkpoints in order to actively avoid immune mediated tumor lysis^[15–19]^. This research has highlighted the critical role that the immune system plays in controlling tumor growth and the importance of reversing immunosuppressive mechanisms. This review will focus on the mechanisms of action of antibodies that inhibit CTLA-4 and programmed cell death protein 1 (PD-1), encompassing therapies that have already been approved by the FDA and others currently in development^[2]^.

## Immune checkpoint inhibitors

**Fig.1:**
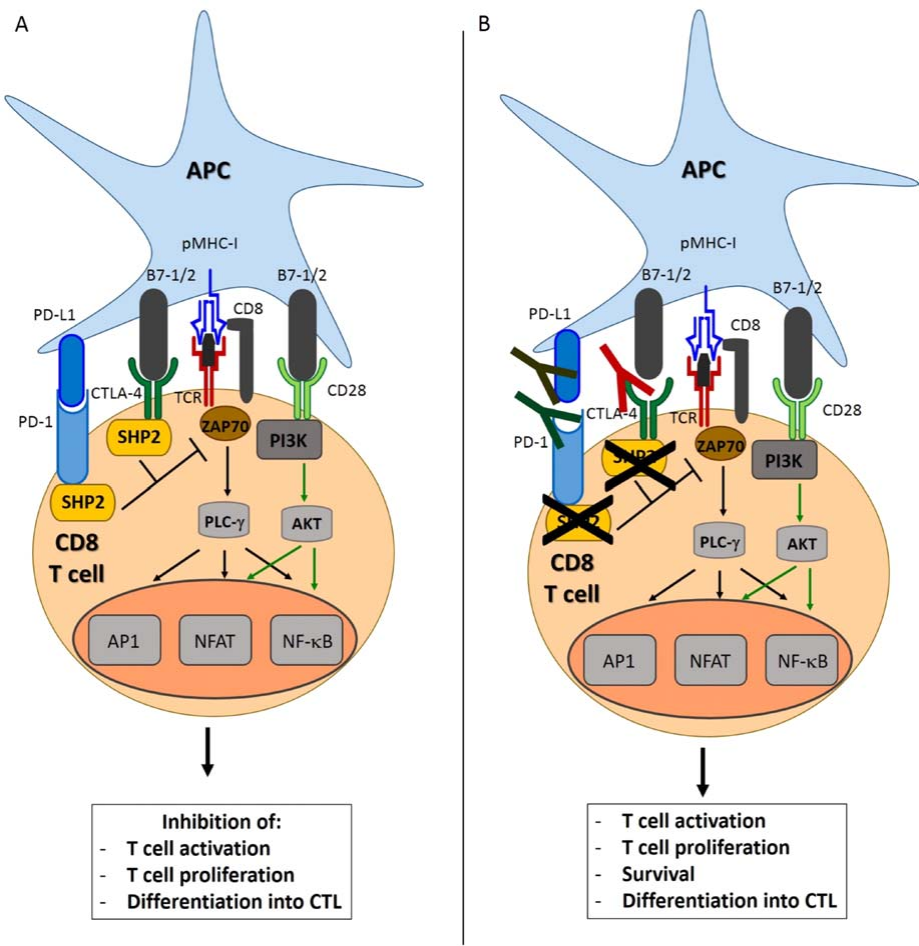
Rationale for the use of immune checkpoint inhibitors in cancer therapy.

The inhibition of immune checkpoints using specially designed checkpoint blocking monoclonal antibodies (mAbs) such as CTLA-4 and PD-1 play an increasingly important role in the treatment against a growing number of malignancies. CTLA-4 attenuates the early activation of naïve and memory T cells through interactions with its ligands B7-1 (CD80) and B7-2 (CD86) (***Fig. 1A***). PD-1 is an receptor expressed on the surface of activated mature T cells, activated NK cells, B cells, monocytes and multiple normal tissues and plays a crucial role in the maintenance of peripheral tolerance^[20–21]^ (***Fig. 1A***). In contrast to CTLA-4, PD-1 acts via interactions with its ligands PD-L1 (also known as B7-H1 or CD274) and is involved mainly in T cell activity modulation in peripheral tissues as well as providing a major immune resistance mechanism within the tumor microenvironment. Cells expressing high levels of PD-L1 may include tumor cells, T cells, APCs, epithelial and hematopoetic cells types among others^[22–25]^. PD-L2 (also known as B7-DC or CD273) is mainly expressed by APCs^[26–28]^.

The presentation of antigenic peptide on the MHC class I molecule to the CD8 T cell is the first step in the induction of an effective immune response with generation of tumor-specific CTLs. In addition to the recognition of pMHC-I by T cells via TCR, induction of primary T cell response requires co-stimulation of the T cell by interaction of B7.1/2 co-stimulatory molecules on the APC with CD28 on the T cell. This interaction results in downstream signaling that leads to T cell activation and further differentiation into CTLs. Upon activation, T cells express the surface proteins CTLA-4 and PD-1, which bind to B7-1/2 and PD-L1, respectively on the surface of APCs. 

Cytotoxic T cells (CTLs) are considered the backbone of immune response against tumor^[29]^. To recognize and eliminate tumor cells, CTLs require two activating signals. The early immune response, occurring mainly in the lymph nodes is known as the "priming" phase and requires two signals for T cell activation. In the first signal, CD8+ T cells recognize antigenic peptides presented by the major histocompatibility complex (MHC) class I molecules on the surface of cancer cells through their T cell receptor (TCR). The second signal, known as the "costimulatory signal" completes primary T cell activation and is achieved through binding of the T cell costimulatory receptor CD28 with the two costimulatory ligands on APCs; B7-1 and B7-2^[28–30]^. This leads to downstream signaling and T cell activation and further differentiation into CTLs (***Fig. 1A***). Of note, CD8 T cells require a third signal, along with antigen and costimulation, to make a productive response and avoid death and/or tolerance induction.

Following activation, CTLs express surface protein receptors, CTLA-4 and PD-1 which function as immune checkpoints. Under usual conditions, the binding between CTLA-4 and B7-1 or B7-2 counteract the costimulatory effects of the CD28 on T cell activation preventing T cell overactivity, as CTLA-4 binds at higher affinity (20 times more)^[30]^. This balance between inactivity and activity control CTL activity and thus CTLA-4 acts as a negative regulator of T cells^[31]^. Reports have shown that an important mechanism of tumor evasion is the upregulated expression of CTLA-4 on T cells with the help of TGF-β, enabling cancers to evade the immune effects of CTLs^[32–35]^.

Similarly, the engagement of PD-1 on a T cell with PD-L1 on the tumor cell surface inhibits T cell function and activation. The PD-1 mediated dysfunction of T cells is thought to be due to a number of mechanisms. Following T cell activation, PD-1 binds to PD-L1 on the surface of APCs and this induces T cell apoptosis, anergy, exhaustion or IL-10 production. (***Fig. 1A***) Further, PD-L1 may act as a barrier to protect tumor associated PD-L1 from CTL lysis^[36–37]^. Additionally, murine models have demonstrated an interaction between PD-L1 and B7-1. B7-1 may be expressed on activated APCs and T cells and may send out inhibitory signals when bound to PD-L1. Tumours and chronic infections can exploit this pathway to downregulate T cell–mediated immunity against tumors and pathogens^[38–39]^. In particular, PD-L1 and to a lesser extent, PD-L2 are expressed on many tumors, including urothelial, colon, pancreatic, gastric cancers, ovarian, breast, cervical as well as melanoma glioblastoma and NSCLC^[28, 40–48]^.

Thus, numerous studies have shown that CTLA-4 and PD-1 have an important role in mediating immune evasion in the tumor microenvironment. The administration of mAbs blocking CTLA-4, PD-1 or PD-L1 allows for the generation of a sustained and specific CTL response capable of tumor cell lysis (***Fig. 1B***)^[20, 26, 28]^. Clinically successful anti-CTLA-4 antibodies blocking this inhibitory signal such as ipilimumab and tremelimumab have been developed which amplify and prolong an anti-tumoral response^[49–50]^.

### Preclinical data

#### CTLA-4 blockade

Following in vitro studies that supported CTLA-4 as a key checkpoint molecule in the antitumor immune response, anti-CTLA-4 blocking antibody therapy was initially tested in numerous animal models including breast^[51]^, prostate^[52]^, lymphoma^[53]^, colon^[54]^ and melanoma^[55]^. The first study, carried out by Alison and colleagues demonstrated that CTLA-4 blockade enhances the anti-tumor immune response^[31]^. Although this efficacy was limited to a few cancer cell lines that only responded to CTLA-4 when combined with a transduced granulocyte-macrophage colony-stimulating factor (GM-CSF) producing cellular vaccine^[51, 56]^. These findings suggested that CTLA-4 blockade could result in significant anti-tumor activity through stimulation of the endogenous antitumor response through enhancement of naturally or vaccine-induced tumor-specific T cells. Further, in the case of poorly immunogenic tumors, which have a limited endogenous immune response, the combination of CTLA-4 antibody with a vaccine has the potential to establish an immune response to hinder tumor growth and lead to tumor regression in certain cases^[2, 28]^.

These studies have paved the way toward CTLA-4 blockade in human clinical trials. A significant phase III trial was published in 2010 for ipilimumab^[57]^ which together with tremelimumab^[58]^ are the two most clinically successful anti-CTLA-4 mAbs^[50]^. Ipilimumab was found to have a significant increase in survival, for patients with previously treated unresectable metastatic melanoma and was the first drug in this class approved by the FDA for use as first or second line therapy for advanced melanoma^[50, 59–61]^.

Further, as well as enhancing overall survival, ipilimumab treatment was associated with a durable response (>2.5 years) with the potential to achieve long-term control of disease in a significant proportion (15-20%) of individuals^[49–50]^. The median duration of response was two years, compared with 4–8 months for chemotherapy regimens and oncogene-targeted therapy^[62]^. The results showed that stable patients at ≥24 weeks were followed up and continued to be stable beyond 48 weeks. Improved durability was associated with improved survival outcomes with one year survival at 42% and 2 year survival at 14%^[63–64]^. Considering the advanced inoperable stage of disease in this patient group, this outcome is encouraging^[49–50, 65]^. These durable responses suggest lasting adaptations in the immune system, supporting the belief that immunomodulating therapy may alter the patient’s intrinsic tumor-specific T cell function^[64–65]^.

#### PD-1 blockade

The role of PD-1 as an important regulator of immunity within the tumor microenvironment through inhibition of T cells has been shown^[60, 66–67]^. It was predicted that PD-1/PD-L1 blockade would have a greater anticancer effect than CTLA-4 inhibitors with fewer unwanted side effects due to the selective immunosuppressive signals delivered by cancer cells^[2, 20]^. Effective antitumor T cell responses have been shown by testing PD-1 blockade together with GM-CSF in murine models such as CT26 colon carcinoma, murine B16 melanoma and pancreatic ductal adenocarcinoma models^[68–69]^. Numerous clinical trials have therapeutically exploited the PD-1/L1 pathway to considerable effect, with durable response rates between 20% to 50% in multiple types of cancer^[21, 60]^. These successes led to FDA approval of the anti-PD-1 antibodies, pembrolizumab (humanized IgG4, Merck) followed by nivolumab (fully human IgG4, Bristol-Myers Squibb, Ono Pharmaceuticals) in 2014, for patients with advanced melanoma who had not responded to anti-CTLA-4^[70–72]^.

Multiple trials have demonstrated that blockade of the PD-1/L1 pathway has effective anti-tumor activity in a number of different malignancies including bladder cancer^[73]^, breast cancer^[66, 74]^, colorectal cancer^[60, 65–67, 75]^, diffuse large B cell lymphoma^[76]^, follicular lymphoma^[77]^, gastric cancer^[66]^, head and neck squamous cell carcinoma^[74]^, Hodgkin’s lymphoma^[78]^, melanoma^[79–84]^, ovarian cancer^[66, 74]^, non–small cell lung cancer (NSCLC)^[8, 60, 65–66, 85–88]^, pancreatic cancer^[8, 27, 66, 74]^, renal cell carcinoma (RCC)^[60, 74, 89]^, prostate cancer^[60, 65]^, sarcoma^[74]^, small cell lung cancer (SCLC) and uterine cancer^[74]^. Further trials are investigating anti-PD-1/L1 administration in other cancers such as lung [90], bladder^[91–93]^, renal cancers^[74, 94]^, breast^[95–96]^ and chemotherapy-refractory Hodgkin disease^[78]^.

### Toxicities

CTLA-4 and PD-1 inhibitors are the most clinically successful checkpoint inhibitors nevertheless, there are a number of concerns including autoimmunity, unique adverse effects and toxicity related to checkpoint blockade mAbs^[97]^. Although direct comparisons have not been carried out, clinical response levels and toxicities are generally consistent between PD-1 and PD-L1. Due to the role of the PD-1 pathway in the maintenance of self-tolerance, the inhibition of this pathway can cause problems, resulting in adverse immunologic responses termed "immune related adverse events" (IRAEs). IRAEs may cause toxicity to tissues and organs which are usually protected by the immune system, resulting in autoimmune-like diseases and inflammation.

However, the most common side effect of PD-1 blockade is fatigue which is not necessarily a limiting factor in treatment duration and does not require medical treatment^[84]^. Other common side effects associated with CIs were decreased appetite (12%) and diarrhea (10%)^[60]^. However, less frequently observed toxicities can occur in pulmonary (inflammatory pneumonitis), endocrine, mucocutaneous and renal (interstitial nephritis) sites and even immunologically privileged sites such as the eye resulting in damage. These events are rare and may be life threatening such as the case of inflammatory pneumonitis, requiring cessation of therapy and treatment with immunosuppressants such as corticosteroids^[98]^. Interestingly, clinical responses persist despite treatment cessation and immunosuppression which suggests that the ideal duration of PD-1 checkpoint blockade has yet to be determined^[99]^.

In one trial 60% of patients treated with anti-CTLA-4 experienced adverse events of which 10%-15% were classed as severe (grade 3/4)^[50]^. IRAEs are less frequent in anti-PD-1 treated patients than in those treated with CTLA-4 blockade (13.3% as opposed to 19.9% in anti-CTLA-4 treated patients) leading to approval of anti-PD-1 treatment as first line for advanced melanoma in the USA and the EU^[2, 98]^. Understanding the adverse effects associated with checkpoint blockade as well as having effective treatment plans for their management are crucial to optimise the efficacy of anti-PD-1 and anti-CTLA-4 therapy.

### Neoantigens

Questions still remain about the degree to which individual host and tumor characteristics determine therapeutic responsiveness and whether these can be used to predict durability and responsiveness. Whole-genome sequencing of tumors has revealed that growing tumors acquire hundreds of somatic tumor specific mutations, which form new antigens designated "neoantigens" which have been seen in mouse tumor models and in CTLA-4 and PD-1 treated patients^[100–102]^.These neoantigens are key determinants in the response of patients to PD-1 and CTLA-4 checkpoint immunotherapy^[102–103]^. Despite neoantigen specific T cells being generated in growing tumors they are unable to produce an effective antitumor immune response. However, several studies have shown that neoantigen specific T cells were reactivated following CIs administration and formed an antitumor response^[101–103]^. The genomics of individual tumors therefore goes some way in explaining the variable responses among patients who have undergone CIs treatment.

### Combination therapy

Notably, preclinical studies of anti-CTLA-4 and anti-PD-1 mAb combinations demonstrated promising results in a range of cancers^[92, 104–105]^. The first phase I clinical trial, combining ipilimumab and nivolumab was updated in an ASCO annual meeting in 2014 showing a 2 year survival of 79% (objective response rate of 43%) among patients with advanced melanoma. However, combination therapy was shown to have increased adverse effects compared to administration of the agents alone (63% of grade 3/4 toxicities)^[106]^.

Further, a recent phase III study assigned untreated patients (*n* = 945) with metastatic melanoma to combination treatment with nivolumab and ipilimumab. The median progression free survival was 11.5 months in the combination treatment in comparison to 2.9 months for ipilimumab and 6.9 months with nivolumab. The study found that patients with PD-L1 negative tumors responded more effectively to a combination of PD-1 and CTLA-4 blockade (11.2 months) as opposed to nivolumab alone (5.3 months)^[106]^. Similar to the 2014 study, treatment related toxicity was higher in the nivolumab-plus-ipilimumab group (55%) as opposed to nivolumab (16.3%) or ipilimumab (27.3%) monotherapy^[82, 106]^. Treatment related adverse events in the combination therapy group are consistent with side effects seen in previous trials^[50, 64, 84]^ and were managed primarily with immune-modulatory agents. Thus, despite the higher incidence of adverse effects in the combination group, the toxicity profile is consistent with anti-CTLA-4/PD-1 monother- apy^[104–105]^.

### Co-stimulatory molecules

Similar to immune checkpoint molecules, agonistic antibodies for co-stimulatory pathways such as CD137 (4-1BB), CD27, *OX40* are showing promise as they augment T cell activation, and therefore may have a role in the antitumor T cell response^[99]^. A CD137 agonist antibody was tested in combination with anti-PD-L1 antibody in a murine breast cancer model which overcame resistance to immune mediated rejection and showed improvements in T cell immunity in other mouse models^[107–108]^. Based on combined treatment with agonistic anti-OX40 antibodies and anti-CTLA-4 antibodies, which induced tumor regression and improved survival^[109]^, further early phase trials investigating combinations of OX40 and PD-L1 and OX40 and CTLA-4 are currently underway in advanced solid tumors (NCT02221960 & NCT02205333). Subsequent trials in solid tumor models have been tested; OX40 and anti-CTLA-4 in ovarian carcinoma (ID8), prostate cancer (TRAMP1), anti-CD137 and CTLA-4 blockade in MC38 colon cancer and GL261 glioblastoma, demonstrating synergy between CD137, PD-1, and CTLA-4^[109–113]^. Based on these promising results, two current phase I/II trials are investigating the combination of anti-PD therapy with anti-CD137 in advanced solid tumors (NCT02554812 and NCT0217-9918).

## Conclusion

The introduction of CIs in the arsenal of immunotherapeutics against cancer has ushered in a new era in the treatment of many cancers. Unprecedented responses have been seen among patients with advanced cancers including melanoma, lung, bladder, RCC and Hodgkin’s disease, treated with anti-CTLA-4, PD-1/L1. However, only less than 25% of patients treated with these agents have got benefit. The future of immunotherapy depends upon identifying and developing ideal combinations of immunotherapies in order to optimise and enhance the efficacy of treatment, as well as achieving a durable anti-cancer effect. More research needs to be conducted into immune checkpoint combination approaches based on individual tumor genetics to be able to predict responses to treatment and increase the number of patients that respond to therapy. Although many challenges still remain, there is a sense of hope that checkpoint inhibitors have heralded a new era in the treatment of many cancers. Combining immune checkpoint antibodies with other immune-stimulating agents such as conventional drugs, targeted agents and most promisingly tumor-targeted oncolytic virus, may open a new avenue for cancer patients in which a durable clinical benefit can be achieved.
